# Treatment of patients with HTLV-1-associated myelopathy with methotrexate

**DOI:** 10.1186/1742-4690-11-S1-P33

**Published:** 2014-01-07

**Authors:** S Ahmed, A Adonis, S Hilburn, MA Demontis, A Fedina, J Haddow, C Gabriel, S Fidler, GP Taylor

**Affiliations:** 1 National Centre for Human Retrovirology, St Marys Hospital, London, UK; 2 Section of Infectious Diseases, Imperial College, London, UK

## Introduction

The lifetime risk of developing HTLV-1 associated myelopathy (HAM) is 0.25-3%. The main pathological feature is an immune-mediated response leading to chronic inflammation of the spinal cord. The optimal long term treatment has yet to be determined although clinical improvement with ciclosporin has been shown in a pilot study. Methotrexate, commonly used for autoimmune diseases, was introduced for the treatment of HAM at the National Centre for Human Retrovirology, London, UK as an alternative to ciclosporin.

## Methods

A retrospective study of patients with chronic HAM treated with methotrexate. Primary outcomes are clinical both objective and subjective using routine clinic data. Secondary outcomes virological/immunological. Methotrexate was prescribed weekly at an initial dose of 7.5mg and a maximum dose of 15mg with weekly folic acid supplementation.

## Results

Duration of methotrexate 4 – 59 weeks. Generally well tolerated. Four haematological toxicities all grade 1/2 and three liver toxicities two grade 3/4 - one treatment discontinuation due to asymptomatic liver toxicity. Objective measure – Compared with baseline -57.39s (18.8-120) mean 10m timed walk improved within 4 weeks 34.54s (16.8-87), p=0.0151. The overall trend shows persistent improvement.

Subjective- Pain as measured on a Visual Analogue Scale improved over the duration of treatment with the greatest improvement occurring within the first 4 weeks of treatment. Virology and Immunological. T-cell activation markers, Th1/2 cytokine data and HTLV viral load data will be presented.

## Conclusions

These preliminary data suggest that methotrexate may be beneficial for some patients with HAM and is a suitable candidate for a randomised controlled study.

